# A Pediatric Supracondylar Fracture with Bilateral (Medial and Lateral) Pillar Comminution–A Recommendation for a New Stable Pin Configuration for a Highly Unstable Fracture

**DOI:** 10.3390/pediatric18010015

**Published:** 2026-01-21

**Authors:** Lara Marie Bogensperger, Sandeep Patwardhan, Stephan Payr

**Affiliations:** 1 Section of Pediatric Trauma Surgery, Department of Trauma Surgery, University Clinic of Orthopedics and Trauma Surgery, Medical University of Vienna, 1090 Vienna, Austria; n11807478@students.meduniwien.ac.at; 2 Department of Trauma Surgery, University Clinic of Orthopedics and Trauma Surgery, Medical University of Vienna, 1090 Vienna, Austria; 3 Department of Pediatric Orthopedics, Sancheti Institute, Pune 411005, Maharashtra, India; sandappa@gmail.com

**Keywords:** pediatric supracondylar fracture, medial comminution, lateral comminution, closed reduction, K-wire fixation

## Abstract

The management of supracondylar fractures in children remains a challenging area of orthopedic practice. Medial comminution is a recognized complication that can result in unstable fracture patterns, which can pose challenges in diagnosis and management. However, when anticipated surgical treatment with an additional medial K-wire is administered, stable fixation is typically ensured. However, an additional radial comminution poses several challenges for reduction, alignment assessment, and pin configuration for stable fixation, as presented in this case. This case report presents a fracture pattern of a Gartland type 3 fracture with medial and lateral comminution that has not been sufficiently described previously and illustrates an effective pin configuration that has yet to be theoretically described.

## 1. Introduction

This injury accounts for up to 48–78% of all fractures of the pediatric elbow joint [1,2,3]. In the contemporary medical context, pediatric supracondylar fractures are widely acknowledged as a particularly challenging type of fracture, particularly in cases of displacement or severe concomitant injuries [4]. Medial comminution is a relatively well-described issue. However, if not detected or managed adequately, it can lead to devastating results, necessitating revision surgery, as is the case for cubitus varus [5,6]. When aware of this issue, the instability resulting from a medial comminution can be effectively addressed with a crossed K-wire configuration or additional medial pin when treating primarily with a lateral pin configuration. In such cases, the medial pin is imperative for achieving the requisite stability [7]. In cases of medial comminution, accompanied by the preservation of the lateral pillar, the conventional reduction and alignment evaluation, in conjunction with the configuration of pins, remains a manageable procedure, as outlined in the extant literature [5,6,7]. In the event of the lateral pillar also being comminuted, the appropriate course of action remains to be determined. This case presentation of a 3-year-old girl with a Gartland type 3 fracture with medial and lateral comminution illustrates an effective treatment and pin configuration for a fracture pattern that has not yet been sufficiently described.

## 2. Case Presentation

**Figure 1:**
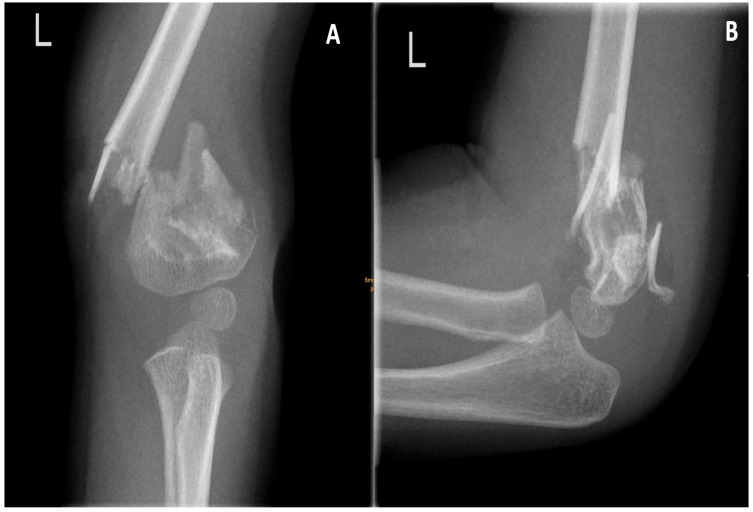
Images of the fracture. (**A**) A.p. image shows complete dislocation, lateral and medial comminution. (**B**) Lateral image also depicting multiple fragments.

A 3-year-old girl fell from a monkey bar on the outstretched hand at approx. 2 p.m. and was transferred to a nearby hospital. The X-ray obtained revealed a supracondylar humerus fracture, which is consistent with a Gartland type 3 fracture. A Gartland type 3 fracture is characterized by a complete dislocation in both views (Figure 1). Additionally, a medial and lateral comminution was observed. The injured extremity was splinted and transferred to a tertiary hospital with a pediatric surgery department. Due to the emergence of inquiries regarding the appropriate approach for addressing this fracture pattern, the patient was referred to our clinic for definitive treatment. The patient arrived at the department at 7 p.m., and the surgical team prepared her for closed reduction and percutaneous pinning due to massive dislocation. She was prepared for immediate surgical intervention at 9:15 p.m. Initially, the patient exhibited a pucker sign accompanied by a concomitant hematoma on the medial side. At this juncture, the degree of swelling remained moderate, with no indications of compartment syndrome, vascular compromise, or neurological impairment. Prior to the surgical intervention, the patient was administered a preoperative antibiotic regimen consisting of 500 mg of cefuroxime. The surgical intervention was executed through the utilization of the aforementioned arm board technique, which was performed under the administration of general anesthesia [8]. The subsequent section delineates the procedural approach and the stabilization process.

Positioning

The patient was positioned supine, with the affected extremity placed on the arm board, which is a length-adjustable, radiolucent device. In this case, the arm board is a carbon model. The arm board was terminated at the level of the fracture site, and the extremity was fixed proximally at this level with a broad hypoallergenic adhesive tape. The distal fragment was maintained in a free position to facilitate reduction and pinning. The image intensifier was positioned parallel to the head end of the table on the same side as the injured limb. Then, the sterile area from the fingers to arm board was draped.

Reduction

The initial implementation of the milking maneuver was executed for the purpose of releasing the proximal fragment from the brachioradialis muscle. This indication was determined by the presence of the pucker sign and the initial a.p. radiograph. The elbow was extended and the C-arm was used in a.p. to check coronal alignment (Varus and valgus malalignment, mediolateral translation) and whether the fracture was at length. Then image intensifier rotated for lateral viewing to check rotational displacements. An adequate reduction in lateral view was achieved by gentle longitudinal traction, minimal pressure on the distal fragment with the thumb and flexion of the distal fragment. The acceptability of reduction was confirmed with the C-arm in anteroposterior and lateral views by rotating the C-arm 90° without moving the extremity.

Pinning

**Figure 2:**
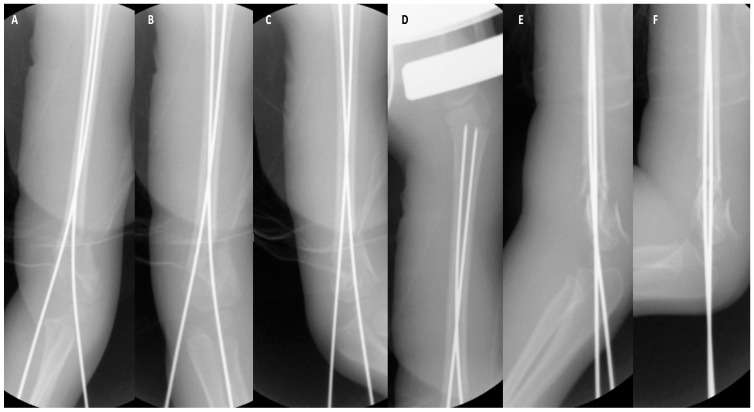
Images after closed reduction and K-wire insertion. (**A**) A.p. image. (**B**) Internal rotated to illustrate adequate reduction in medial column. (**C**) External rotated to illustrate adequate reduction in lateral column. (**D**) K-wire placement proximal ending before the physis. (**E**) Lateral image. (**F**) Lateral with slight extension to illustrate stability.

The first lateral pin (1.8 mm × 280 mm) was the olecranon fossa pin with tetracortical purchase passing from the lateral condyle across the olecranon fossa, then intramedullary up the proximal humeral physis. The second pin (1.8 mm × 280 mm) was put from the medial in a cross-pin fashion from the medial epicondyle region through the medial column intramedullary, also up to the proximal humeral physis. In order to minimize ulnar nerve injury, the pin was inserted at a 45° flexion and from an anterior entry point. Subsequent to the second pin, the stability and alignment of the fracture were examined in a.p. and lateral positions. Further a.p. images were obtained, including those depicting internal and external rotation, as well as lateral images in extension and flexion positions (Figure 2).

Radiological and clinical assessment

The reduction and alignment were checked radiologically in a.p., lateral, 45° internal and external rotation. In a.p., the reduction was assessed as appropriate since the alignment was congruent and no caliber leap was obvious. In lateral, the reduction was assessed as appropriate since an hourglass sign was detectable, the anterior humeral line was aligning with the capitellum in an acceptable fashion, and also, no caliber leap was present. Fracture stability was further verified under the C-arm during movement.

Clinically, ROM was acceptable and a physiological valgus (5–7°; the uninjured limb was therefore examined beforehand) was restored.

Postoperative cast application

Postoperatively, a slit upper arm cast was applied in forearm supination and in 80° flexion. The latter was intended to mitigate the risk of compartment syndrome. Capillary refill was finally checked again and was under 1 s.

Postoperative care and outcome

**Figure 3:**
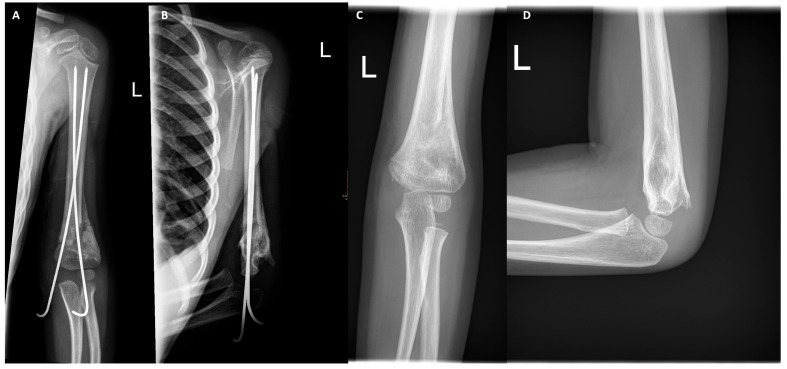
Post-op X-ray after 4 weeks before K-wire removal and 17 weeks at full ROM. (**A**) First X-ray four weeks post-op before K-wire removal (a.p.). (**B**) Lateral image. (**C**) A.p. image illustrating bony healing 17 weeks post op corresponding to free ROM and restored clinical axis. (**D**) Lateral image.

Patient was released from hospital 24 h postoperatively. All subsequent appointments were scheduled in the outpatient department. The cast was applied for a period of four weeks, and a single cast change was performed after a duration of two weeks. X-ray imaging was conducted following the removal of the cast at week four, revealing adequate bony healing across all four cortices (Figure 3). On this day, pin removal was also carried out in a semi-sterile room without additional anesthesia. After removal, an iodine ointment and a bandage to be removed after 2 days to by the parents was applied. Subsequent to the removal of the pin, movement was permitted. First, clinical evaluation was 2 weeks after pin removal. ROM at that time point was 0-0-90° and a correct clinical axis comparable to the uninjured extremity was observed. During FUPs, ROM improved steadily and ROM on the affected side was documented as being equivalent to that of the uninjured side during a FUP visit after 17 weeks postoperation. No functional and neurological deficits were present. There were no complaints from the patient or her family at that time point.

## 3. Discussion

The presented case report details a surgical treatment and fixation option that has been proven to be both adequate and relatively easy, with a justifiable basis in the overall excellent outcome for a fracture pattern of a pediatric supracondylar fracture that has not yet been sufficiently described. Despite the presence of bilateral comminution, radiological signs such as a caliber leap, an hourglass sign, and an anterior humeral line, in conjunction with radiological and clinical valgus, were employed to assess the adequacy of reduction in this particular fracture type. Achieving exact anatomical reduction may be unfeasible. Therefore, it is imperative to understand the criteria for adequate reduction and to determine the extent to which residual dislocation or angulation is still adequate. For this assessment of adequacy, the combination of the aforementioned radiological and clinical features was applied and appropriate. In this case, it can be argued that the reduction is not optimal, particularly in the sagittal plane. This is due to the anterior humeral line not clearly crossing the capitellum between the anterior and middle thirds. This may result in a clinically restricted flexion. The authors are aware that the anterior humeral line does not clearly intersect the capitellum. However, the least amount of reduction by “scratching” it in the anterior aspect was considered sufficient. From a personal standpoint, it is imperative to take into account the additional healing bone, which appears to be enlarged. This may consequently lead to alterations or complications in the precise evaluation of the anterior humeral line. It must be acknowledged that this is merely speculative, yet it is reasonable to hypothesize that the patient’s age (3 years) and the remodeling potential that remains until the age of 5 are the most probable factors contributing to the success of this reduction. The reduction, however, was initially accepted by the senior surgeon and should not be attributed to the application of K-wires.

The primary challenge posed by bilateral comminution is that conventional cross-pinning recommendations are often not applicable in such cases. This is due to the fact that the anticipated exertion points would be situated precisely within the comminuted zone [9]. The senior author considered this fracture type with bilateral comminution to be a high metaphyseal fracture and therefore immediately decided to go for the crossed intramedullary K-wire configuration as used in this case, which is, however, only theoretically suggested in the literature [7]. A review of the extant literature reveals that, when considering this fracture type with bilateral comminution as a high metaphyseal fracture, there are only a few practical and one theoretical recommendation [7,9,10,11]. The practical solutions that are described herein include the crossed K-wire configuration for high metaphyseal fractures but without comminution and the use of ESIN from the proximal or distal end [9,10,11]. However, the utilization of ESIN, irrespective of the insertion point, appeared to be suboptimal for several reasons. From proximal, the senior author’s hesitation to consider ESIN as a suitable treatment option was driven by the anticipated severely limited bone stock in the distal fragment, particularly in a three-year-old patient and with bilateral comminution. It is evident that entering through the distal part would result in traversing the growth plate of the distal humerus. This suggests that the procedure is inadequate at this age, considering the remaining time of growth in the distal humerus. In both scenarios, the utilization of ESIN would have entailed the establishment of a more expansive operation site, consequently resulting in the formation of more pronounced scars. This would have yielded an esthetically suboptimal outcome in comparison to the minimal pin marks that have been observed in the use of K-wires. Furthermore, ESINs would have requested a secondary surgical intervention (implant removal), given that ESINs are typically positioned beneath the skin surface, in contrast to K-wires, which can be left above the skin surface and simply extracted when appropriate. This approach would eliminate the need for a second round of anesthesia, surgery, and, in most cases, stitch removal. After all, ESIN might have facilitated the achievement of a superior sagittal alignment (anteflexion angle) due to its bent tip. However, the presence of bent tips surrounded by comminution, particularly in the dorsolateral and dorsomedial regions, may have indicated a potential risk of compromised stability. Conversely, the intramedullary K-wire configuration that was employed in this case offers several advantages that were deemed to be sufficient and advantageous. The senior author postulated that a significant benefit of the aforementioned technique would be the capacity to address the distal fragment directly, thereby enhancing its stability. Furthermore, the selection of the entry point at the capitellum ensured the engagement of the maximum bone stock in the distal fragment [12]. Moreover, the capacity to utilize the lateral pin as a tertacortical pin is indicative of enhanced stability. Furthermore, when this pin configuration is performed with long K-wires, which are brought up to the proximal physis, the comminuted area can be considered to be sufficiently overcome. The final advantage of utilizing K-wires is that it results in a considerably reduced operation site. When K-wires are positioned above skin level, minimal scarring can be achieved, thereby circumventing the need for a subsequent operation to remove the implants. The technical, administrative, and patient-centered advantages and disadvantages of this technique in comparison to other methods can be summarized as follows. Technical: Maximum stability and contact in the distal bone stock; stable bridging of the comminuted area by maximum distal and proximal hold; least amount of endangering the physis by maximum benefit/stability; absolute minimum scarring (no incisions only pin marks), therefore, best cosmetic outcome; no second anesthesia and operation necessary for implant removal; No administrative challenges were faced during this case and therefore cannot be discussed in relation to other methods. Patient-centered aspects would include the excellent cosmetic result and the avoidance of a secondary surgery.

This crossed intramedullary K-wire fixation is not adequately delineated in the extant literature; consequently, several additional considerations were necessary. This is particularly salient in the context of achieving optimal stability and protecting the growth plates. Initially, K-wires were terminated proximally, precisely at the point preceding the proximal humeral physis, with the objective of attaining optimal stability while preserving the physis. In order to mitigate the potential for iatrogenic injuries to the distal physis, a series of precautionary measures were implemented. Taking into account the recommendations for stability and appropriate pin size (two 2 mm pins are more stable than three 1.6 mm pins), the development of the pediatric elbow (90% of growth is completed by the age of five) and the recommendations for secure physeal anchorage (max. size 1.8 mm), a diameter of 1.8 mm was selected for the K-wires used [12]. Prior to this case, there were no extant references for bilateral comminution in pediatric supracondylar fractures, and the use of an intramedullary crossed K-wire fixation method had only been described theoretically.

## 4. Conclusions

This case report is noteworthy for its comprehensive description of a highly unstable fracture pattern in a pediatric supracondylar fracture. It also demonstrates the efficacy of crossed intramedullary K-wires in treating such fractures, thereby establishing their applicability to high metaphyseal fractures in general.

## Data Availability

The original contributions presented in this study are included in the article. Further inquiries can be directed to the corresponding author.
